# Transfer of patients’ tibiofemoral kinematics and loads to a six-degree-of-freedom (6-DOF) joint simulator under consideration of virtual ligaments

**DOI:** 10.1038/s41598-025-95400-4

**Published:** 2025-03-27

**Authors:** Paul Henke, Leo Ruehrmund, Jessica Hembus, Sven Krueger, Rainer Bader, Maeruan Kebbach

**Affiliations:** 1 Department of Orthopaedics, Rostock University Medical Center, Rostock, Germany; 2 Research and Development, Aesculap AG, Tuttlingen, Germany

**Keywords:** Total knee replacement, Biomechanical testing, Six-degree-of-freedom joint simulator, Activities of daily living, Ligament apparatus, Biomedical engineering, Ligaments

## Abstract

Preclinical testing of total knee replacements (TKR) is crucial for evaluating new implant designs. Dynamic experimental testing focus mostly on level walking and squats, failing to represent a full range of daily activities. Moreover, the contribution of the ligament apparatus is often simplified. Therefore, this study transferred five daily activity load cases—level walking, downhill walking, stair descent, squat, and sit-to-stand—onto a six-degree-of-freedom (6-DOF) joint simulator with a cruciate-retaining bicondylar TKR and a virtual ligament apparatus. Forces and kinematics were based on telemetric data from an ultra-congruent TKR. The resulting kinematics, kinetics, and tibiofemoral contact surfaces were evaluated. Additionally, variations of the virtual ligament apparatus on the joint simulator, e.g. resection of the posterior cruciate ligament (PCL), have been used to assess its influence on the resulting joint dynamics. Results showed that tibiofemoral contact area was more influenced by dynamics than kinematics. Virtual PCL resection shifted the tibia posteriorly (up to 3 mm) and increased abduction (up to 0.5°). Different results were seen across all load cases. The exceptions are the squat and sit-to-stand load cases with similar patterns. Thus, cruciate-retaining TKR can be tested using telemetric data from ultra-congruent TKR, aiding in comprehensive evaluations.

**Supplementary Information:**

The online version contains supplementary material available at 10.1038/s41598-025-95400-4.

## Introduction

In preclinical testing of total knee replacement (TKR), both numerical simulations, e.g., multibody^1–3^ and finite element modelling^4,5^, and experimental tests are applied. The latter are usually represented by test standards, e.g. investigation of the wear behaviour of TKR according to ISO standard 14243-1^6^. This is intended to simulate *level walking*, where the anterior and posterior cruciate ligaments are considered in a simplified way via linear displacement-force and rotation-moment relationships. It is known that knee kinematics differ depending on the activities of daily living (ADLs)^7,8^ which have a significant influence on wear propagation^9,10^. Furthermore, the experimental standards^6^ represent the activity *level walking* with restrictions on the controlled degrees of freedom (DOF). The complex dynamics of the tibiofemoral joint in the patient represents six degrees of freedom (DOF)^11–13^.

For research and development of TKR, kinematics and kinetics and the interaction with the soft tissue are of interest. To overcome the limitations of experimental standards, the use of ADLs based on instrumentally derived data promises more physiological boundary conditions to investigate TKRs. In general, these studies focus primarily on determining the wear behaviour of joint endoprostheses. Reinders et al.^9^ investigated the contribution of the different ADLs to wear propagation. Maag et al.^14^ investigate the impact of using either mechanical or anatomical alignment on the loading conditions and wear of the total knee endoprosthesis during walking. Dreyer et al.^15^ experimentally validated a finite element-based wear and creep model with gait data. In these studies, the ligamentous apparatus or the interaction of the ligamentous apparatus with the TKR system was not taken into account or only in a very simplified form^9^. In the literature, the ligamentous apparatus is generally only used in simplified laxity tests^16,17^, passive flexion^18,19^ or under reduced load profiles^17^. A systematic investigation of ADL loading cases taking into account a ligamentous apparatus under physiological loading conditions has not yet been carried out.

The research group of Taylor et al.^8^ provided comprehensive assessment of the musculoskeletal system (CAMS) with valuable datasets on kinematics and loading of the knee joint during various ADLs of different subjects. The TKR used was the INNEX FIXUC ultra-congruent total knee design (Zimmer Biomet, Winterthur, Switzerland). Important insights were gained through synchronised measurements on six subjects with an instrumented TKR, including kinematics and kinetics with a moving fluoroscopy^8^. The data from these six test subjects were merged into uniform load cases of the standardised test subject “Stan”, according to Dreyer et al.^20^. Based on this dataset, the data derived from the instrumented TKR were used as input for experimental investigations, e.g. joint simulators, to analyse TKR dynamics. However, studies simplified^21^ or neglected^14^ the surrounding ligament apparatus. To overcome this, human specimens were used to consider the ligaments and test different implant designs under consideration of the surrounding ligaments^22–24^. However, studies based on human specimens face limitations regarding the specimens’ availability and individual variability, making reproducibility and parameter variations on the same specimen difficult.

Therefore, our present study aimed to transfer five standardised load cases of the CAMS dataset, namely: *level walking*, *downhill walking*, *stairs down*, *squat* and *sit-to-stand* to the six-DOF joint simulator VIVO™ (Advanced Mechanical Technology, Inc., Watertown, MA, USA) to test a posterior cruciate ligament retaining bicondylar TKR under consideration of the knee ligaments. Tests were conducted with physiological loadings and speeds to investigate the suitability of these load cases for the experimental characterisation of TKR using a six-DOF joint simulator. Further, the extent to which the kinematic and kinetic data can be transferred to other TKR designs should be clarified. Considering a virtual ligament apparatus, the effect of various soft tissue tension on the joint dynamics should also be investigated to show the feasibility of our test approach.

## Results

The VIVO™ joint simulator displays joint kinematics according to Grood and Suntay^25^, which considers the motion of the tibial coordinate system relative to the femoral coordinate system. Forces and moments are interpreted in the origin of the tibial coordinate system according to Grood and Suntay^26^. For example, positive inferior-superior forces lead to joint compressions, and positive anterior-posterior forces will push the tibia anteriorly.

### Reference ligament apparatus

**Fig. 1:**
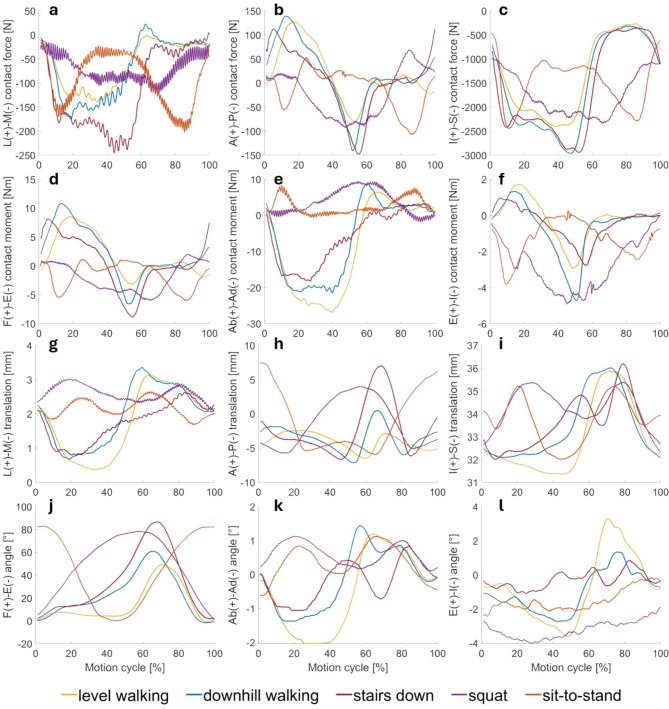
Tibiofemoral kinematics and kinetics during different load cases. Kinematics (**a-f**) and kinetics (**g-l**) for level walking (yellow), downhill walking (blue), stairs down (red), squat (purple) and sit-to-stand (orange) with reference apparatus. (**a**) Lateral-medial L-M contact force, (**b**) anterior-posterior A-P contact force, (**c**) inferior-superior I-S contact force, (**d**) flexion-extension F-E contact moment, (**e**) abduction-adduction Ab-Ad contact moment, (**f**) external-internal rotation E-I contact moment, (**g**) lateral-medial L-M translation, (**h**) anterior-posterior A-P translation, (**i**) inferior-superior I-S translation, (**j**) flexion-extension F-E angle, (**k**) abduction-adduction Ab-Ad angle, (**l**) external-internal rotation E-I angle.

Figure 1 shows the kinematics (A-F), the kinetics (G-K) of all five load cases with the reference ligament apparatus for P.F.C. Sigma. Note that the graphs only show the relevant part of the load case without the additional 15% motion cycle necessary to harmonise the initial and final values. Due to recurring similarities in the kinematic and kinetic results, grouping the level walking, downhill walking, and stairs down into “locomotion load cases” and the squat and sit-to-stand into “standing load cases” is appropriate.

The absolute maximum inferior-superior tibiofemoral contact force (axis load) is highest during *stairs down* and *downhill walking* at 2,943 N and 2,930 N, respectively. The locomotion load cases also show a similar curve for the anterior-posterior forces. At the beginning of the cycle, forces increase from 5 to 18% motion cycle before they fall and reach their minimum at 52–55% motion cycle. The inferior-superior force ranges are highest for *stairs down* and *downhill walking*. All load cases show a minimum at around half of the motion cycle for the external-internal rotation moment, except for the *sit-to-stand* load case, where a maximum occurs instead. The maximum flexion angles vary between the load cases from 49° for *level walking* to 87° for *stairs down*. In general, the load cases start and end at approx. 0° flexion, except for the *sit-to-stand* load case, which is in maximum flexion at the beginning and end of the motion cycle. The locomotion load cases show similar trends regarding the tibial anterior-posterior translation. The anterior-posterior translation decreases to a minimum at 53–65% motion cycle before rapidly reaching its maximum (local maximum during *level walking*) and falling again shortly afterwards. *Squa*t starts at − 4.2 mm anterior-posterior translation and rises to 4.0 mm by the middle of the load case. In comparison, *sit-to-stand* starts in the positive region at 7.5 mm and falls to − 4.0 mm in the middle before both load cases approach their starting values again in the second half of the motion cycle. *Level walking* has the largest range of abduction with a range of 3.2°, followed by *downhill walking* with 2.8°.

### Tibiofemoral contact areas

**Fig. 2:**
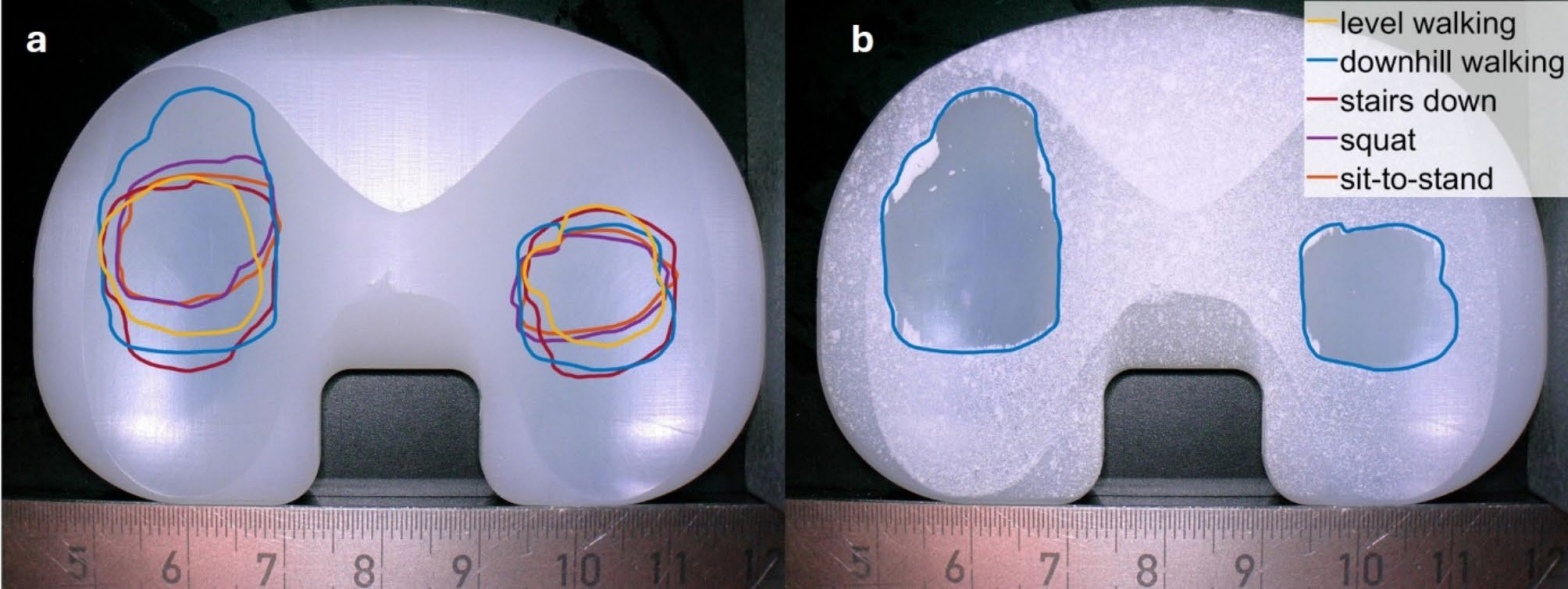
Tibiofemoral contact areas during all five load cases. (**a**) digitally marked contact area for all load cases with reference apparatus (level walking – yellow, downhill walking – blue, stairs down – red, squat – purple and sit-to-stand – orange) on a clean surface insert, (**b**) contact area for downhill walking on an insert with scanning spray used to mark the untouched regions of the insert. The images were taken and processed with the software of the digital microscope VHX-6000 (V 2.8.0.110, Keyence Deutschland GmbH, Neu-Isenburg, Germany) and the contact areas were manually highlighted using Paint.NET (V5.0.1, Washington State University, Pullman, WA, USA and Microsoft Corporation, Redmond, WA, USA, https://www.getpaint.net/).

To analyse the tibiofemoral contact surfaces, the contour of the areas on the tibial insert that showed no residues of the scanning spray was digitally visualised (Fig. 2). A direct comparison reveals that *downhill walking* had the largest contact area, followed by *stairs down* and *level walking*. The contact areas of the standing load cases sit-to-stand, and squat, are very similar in size and position. In all load cases, the contact areas on the medial side are larger and more anterior compared to the lateral side.

### Variations of the ligament apparatus

**Fig. 3:**
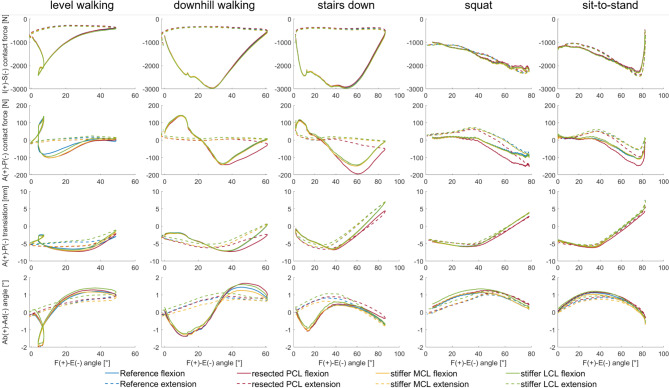
Influence of the ligament apparatus. Inferior-superior forces, anterior-posterior forces, anterior-posterior translation, abduction-adduction angle with respect to the flexion (straight line) and extension (dashed line) angle under consideration of the four different ligament apparatus variations: reference (blue), resected PCL (red), stiffer medial structures (yellow) and stiffer lateral structures (green).

Figure 3 shows the inferior-superior force, the anterior-posterior force and translation, and the abduction-adduction angle of all five load cases for different ligament variations. The variables are shown relative to the flexion angle and categorised into flexion (solid line) and extension movement (dashed line).

Resection of the PCL generally lead to a decrease in absolute inferior-superior forces at a flexion angle of approx. 30–40° for all load cases. The highest percentage change compared to the intact ligament apparatus is found for the *squat* load case. Here, the absolute maximum force is reduced from 2,336 N to 2,257 N, which corresponds to a reduction of 3.4%. Furthermore, PCL resection leads to a higher anterior load, which becomes apparent between 30°-40° flexion angle in the load cases. The *level walking* load case is an exception, where differences are already evident at 10° flexion. Also, the tibia moves more anteriorly during all movements after resecting the PCL. The influence on tibial anterior-posterior translation is highest for *stairs down* with a maximum difference in change of more than 3 mm. In contrast, the tibia does not move more than 1 mm anteriorly in the *squat*. For the load cases, the PCL resection is also linked to increased abduction from a 30°-40° flexion angle, with a maximum change of 0.5° (*stairs down*).

Both the doubling of the stiffness of the medial ligament and lateral ligament structures leads to a slight increase in the inferior-superior forces over the entire flexion cycle. Thus, the stiffening of the lateral structures results in an average of 35 N higher inferior-superior forces, while the stiffer medial side only results in an average of 11 N higher forces. The stiffening of the ligament structures medially and laterally has barely any noticeable effect on the resulting anterior-posterior contact force forces, except for the *level walking* load case. Here, the forces are influenced by medial stiffening similar to PCL resection with force differences up to 34 N. The influence of medial and lateral stiffening on anterior-posterior translation varies for the different load cases. In the load cases *downhill walking* and *stairs down*, for example, an influence is only apparent when the lateral structures are stiffened and only during the extension movement. The greatest changes occurred during *level walking*, whereby the displacement of the medial and lateral structures is accompanied by a 1.3 mm and 1.9 mm higher anterior displacement (at maximum flexion). On the other hand, we cannot observe noteworthy changes for *squat* and *sit-to-stand*.

## Discussion

This study aims to investigate a cruciate-retaining TKR design by transferring five load cases derived from in vivo measurements using instrumented TKR and moving fluoroscopy^8^ to a six-DOF joint simulator under consideration of the ligament apparatus around the tibiofemoral joint. In addition to the different kinetics and kinematics of the load cases, the suitability of these standardised load cases for investigating the soft tissue situation and the resulting joint kinematics and kinetics when applied to a different TKR design, including PCL resection, was analysed. The experiments were performed in real-time and under physiological loads.

The results indicate that the size of the tibiofemoral contact areas depends more on the tibiofemoral loads than on kinematic variables such as AP translation or flexion angle. The high similarity of the *squat* and *sit-to-stand* load cases in kinematics, kinetics and contact areas raises the question of whether both load cases need to be investigated in future experimental and tribological studies. The resection of the PCL led to posterior displacement of the tibia at flexion angles above 30°. In addition, it was also shown to influence varus-varus stability^27^.

All five load cases could be tested successfully using the reference ligament apparatus as there were no unusually high control errors, stumbling or implant dislocations. Only slight vibrations occurred for the medial force, but these did not have any considerable influence on the resulting kinematics. It is to minimise in the future by adjusting the control parameters. At first sight, the five load cases appear to differ greatly in terms of kinematics and kinetics. However, regarding the load cases as a function of the flexion angle, a high degree of similarity between the *squat* and *sit-to-stand* load cases can be noticed, especially if the final phase during sitting (maximum flexion) is disregarded, which has little mechanical and tribological relevance. Based on these results it is up to debate whether it is necessary to consider both standing load cases for future tribological and dynamic investigations.

The similarity between the locomotion load cases *downhill walking*, *stairs down*, and *level walking* in the curves of flexion-extension angle, inferior-superior force and anterior-posterior force can be explained by the fact that all three load cases go through the same sequential movement phases of the stance phase and swing phase^28^. The inferior-superior forces for *squat* and *sit-to-stand* appear to be acting in opposite directions throughout almost the entire cycle, which can be explained by the fact that the movements are nearly 180° phase-shifted to each other. Only the rest phase at the beginning and end of the movement in the *sit-to-stand* load case is absent in the squat load case. The extent of the anterior-posterior translation correlates with the extent of flexion of the load cases because the PCL tenses with increasing flexion, resulting in an anterior translation of the tibial insert.

*Squat* and *sit-to-stand* have the smallest tibiofemoral contact areas, although they have the largest anterior-posterior translation, along with *stairs down*. The contact areas appear to correlate more with the inferior-superior load than the anterior-posterior translation, which also favours the fact that *stairs down* and *downhill walking* have the largest contact areas. Medial contact areas of the load cases are larger than the lateral ones, which is consistent with wear patterns from the literature^29^. 70% o the load is transmitted through the medial condyle and only 30% trough the lateral condyle^30^.

The contact areas of squat and sit-to-stand are very similar. Furthermore, when considering the load cases as a function of the flexion angle, a high degree of similarity between the *squat* and *sit-to-stand* load cases can be noticed, particularly when neglecting the final phase during sitting (maximum flexion). Based on these results, it is up to debate whether it is necessary to consider both standing load cases for future tribological and dynamic investigations. Otherwise, the contact surfaces of the other load cases differ in position and size, which tends to support the consideration of different load profiles in tribological studies. However, it must be noted that the contact areas determined above do not indicate the actual stress on the tibial surface. Without more in-depth investigations, the contact areas shown serve solely as qualitative indication that wear occurs at these locations.

For a detailed categorisation of the respective results, it is important to distinguish between the control variables and the resulting responses i.e. the variables that were transferred to the software of the simulator to actively match them (control variables) and the variables that adjusted passively due to the load case (resulting responses). If the control deviations for one DOF turned out to be minor, this does not automatically suggest the plausibility of the load case, but rather only emphasises the control of the simulator. For instance, the flexion-extension and the external-internal rotation were kinematically controlled based on the *Stan* dataset so that the curves in both datasets are congruent. Although the inferior-superior and anterior-posterior forces were also controlled in the simulator test with the P.F.C. Sigma, they differ from each other in the experiment due to the additional ligament apparatus. Subsequently, the analysed reaction variables, i.e., the variables that are not controlled, are discussed. Firstly, the anterior-posterior translation is of interest. Here it is apparent that the relative trend for all five load cases tends to agree well with the *Stan* dataset but shows an average offset of 9 mm (+/- 2.2 mm) to the posterior (Supplementary Fig. 1–5). This is probably due to the different implant design and geometry, as the virtual ligament apparatus tends to pull the P.F.C. Sigma more anteriorly. The offset in the anterior translation also causes the flexion-extension moment measured in the joint simulator to be considerably lower, as this directly impacts the lever arm of the inferior-superior forces. The medial translation is higher for the load cases with high medial-lateral forces (*downhill walking* and *stairs down*) in comparison to the *Stan* dataset (Supplementary Fig. 2, Supplementary Fig. 5), which may be due to the less congruent implant geometry. The inferior-superior translation of both datasets and the abduction angles tends to be in a similar range. The external-internal moments of rotation of the load cases follow the same course as the *Stan* dataset. However, the amplitude in the experimental test with the P.F.C. Sigma is considerably lower for *level walking* and *downhill walking* (Supplementary Fig. 1, Supplementary Fig. 2) which probably is due to the unconstrained design of the P.F.C. Sigma^31^.

It was shown that variations of the ligaments generally influence the maximum inferior-superior implant load less than the other acting forces. The influence of anterior-posterior loading is higher in relative terms. In the case of PCL resection, this was mostly evident in flexion angles starting from a range of 30° to 40° flexion, which corresponds well with the literature^32^, as the PCL usually begins to tighten in this range. An exception is *level walking*, where a noticeable change in the anterior-posterior force already occurred at significantly earlier flexion angles. This may be because in this load case, the tibia was already displaced far posteriorly at low flexion angles, which also led to tension in the PCL. Interestingly, this was also the only load case in which stiffening the medial structures achieved similar influence on the resulting anterior-posterior forces. For all other load cases, the variation of the medial or lateral structures did not play a considerable role in the anterior-posterior forces and translations. As expected, PCL resection caused the tibia to shift posteriorly during higher flexion angles. As expected, the stiffened medial and lateral ligament structures increased adduction and abduction according to their force direction^33^. Interestingly, at flexion angles above 50°, resection of the PCL shows greater influence on the abduction angle than doubling the stiffness of the lateral ligament structures. This may indicate that at high flexion angles, the PCL not only limits the posterior translation of the tibia but is also involved in the varus-valgus stability of the knee joint^27^.

Some limitations of our present study need to be addressed. First, only one implant was tested, meaning the tests could only be carried out with one implant design (cruciate ligament-retaining). It is unclear whether transferring the test setup (especially at high flexion angles) to other implant designs, e.g. posterior-stabilised TKR is also possible without any restrictions as the load profiles are from patients with an ultra-congruent TKR design. It is assumed that the standardised load cases cannot consider the additional contact occurring at the post-cam-interface. In general, it is a limitation that the initially telemetrically measured in vivo data were based on an ultra-congruent TKR design (with PCL resection) and were applied to a barely congruent design retaining the PCL. Furthermore, only one standardised cycle was used for each load case, which neglects the variability of constantly changing and of variable magnitudes during daily life. Analogous to a previous study^17^, our approach neglected the interaction of the implant with the musculoskeletal system by using input data from instrumented TKR. However, in our approach, the interaction between the implant components and the virtual ligaments of the joint simulator was considered. A further approach could be the use of computer-based simulations to create more realistic contact conditions for specific implant designs^1,14,34^.

The simulator compliance described and published by Kleist et al.^19^ results in an underestimation of the actual applied ligament forces. As the simulator deforms under load, but is not able to detect this deformation with its sensors, the distance between the femur and tibia will be lower than it actually is under higher loads in reality. To address this problem the reference pose was set using the average axial load minimizing this error. Nevertheless, deviations may occur in the respective extremes in load cases with a considerable load range (e.g. downhill walking between − 315 N and − 2,963 N). The virtual ligaments act as point-to-point force elements and are therefore unable to realise wrapping around bones, as this is the case for multiple ligaments in vivo, e.g. the medial collateral ligament^35^. PCL resection and stiffening of the medial and lateral structures were performed for the soft tissue situation variations. The latter procedure has no clinical relevance, as it is impossible to stiffen the structures intraoperatively to this extent. The variations were chosen to test whether the methodology is generally suitable for investigating different soft tissue situations and to better classify the effect of the clinically relevant PCL resection. The results of our present study confirm the feasibility of the proposed methodology to address soft tissue balancing in future studies.

The joint loads presented in the CAMS dataset^8^ were captured in vivo and therefore already contain forces caused by the ligament apparatus of the tested subjects. As we know no approach to calculate the influence of the ligament forces, they were also transferred to the VIVO™ joint simulator, resulting in forces of two ligament apparatus acting in the final experiments, the ones of the in vivo data as well as the ones caused by the virtual ligaments. The comparability with other literature studies may reveal deviation due to the definition of the coordinate systems as shown by Hull^36^ and Ortigas-Vasquez et al.^37^ that different local segment frames may lead to different kinematic patterns. The coordinate systems describing kinematics and kinetics were adapted from previous studies^38–40^ according to ISB recommendations^41^. According to Hull^36^, these recommendations do not necessarily guarantee ideal ‘functional axes’ and therefore kinematic crosstalk errors cannot be ruled out. For future studies, a frame orientation optimisation method^37^ may be used to eliminate possible cross-talk due to non-functional axes.

Various studies have investigated ADLs under physiological loading. Reinders et al.^9^ used in vivo load data to investigate the wear behaviour of TKR for the load cases level walking, stairs up, stairs down, sit-to-stand-to-sit and cycling with peak forces of more than 3000 N (stairs up and stairs down). For level walking, ISO 14,243^6^ specifies peak compressive forces of 2600 N. In the study by Maag et al.^14^, telemetrically measured tibial forces were used to drive a finite element model of the lower limbs, which was used to generate implant- and alignment-specific forces for the ‘level walking’ load case. The data used corresponded to a test subject with a body weight of 1000 N, so that physiological load curves with compressive forces of over 2000 N could be generated for simulator tests. Dreyer et al.^15^ conducted gait tests to experimentally validate a finite element-based wear and creep model, with forces reaching up to 3187 N. With peak forces of 2943 N for stairs down and 2401 N for level walking, the results of this study align well with the existing literature.

There are only few studies on TKR in which the entire movement cycle of these load cases were investigated experimentally under consideration of the ligaments^14,17^. Only the study by Vakili et al.^17^ involved virtual modeling of the soft tissue. In contrast to our study, six different ligamentous apparatus were considered which were determined by previous examinations on human specimens in combination with an optimisation algorithm^16^. However, the acting loads were scaled down to 25%, which made it difficult to estimate the exact influence of the ligamentous apparatus on the resulting kinematics and kinetics as studies on native joints show a dependency between joint loadings and knee kinematics^42,43^.

## Conclusion and outlook

Five load cases derived from in vivo data were successfully transferred to the six-DOF joint simulator for different TKR designs considering nonlinear ligaments. Kinematics and kinetics alongside the tibiofemoral contact areas were evaluated. Furthermore, the effects of various soft tissue situations on the resulting joint dynamics were analyzed through direct parameter changes on the joint simulator. The kinematics and kinetics, as well as the tibial contact areas vary across the investigated load cases. The exceptions are the *squat* and *sit-to-stand* load cases with similar patterns. Resection of the PCL not only affects anterior-posterior translation but also contributes to varus-valgus stability at higher flexion angles. Although the transfer of load cases was successful, alternative methods and techniques need to be developed in the future, e.g. coupling the joint simulator with musculoskeletal simulation models to interact with the acting muscle forces.

## Methods

### Preparation of standardised load cases

In 2022, Dreyer et al.^20^ presented standardised load cases based on telemetrically recorded data from six test subjects from the “CAMS-Knee Project”^8^ which were unified under a standardised subject “Stan”. The standardised load cases include loads from *level walking*, *downhill walking*, *stairs down*, *squat* and *sit-to-stand*, derived from an instrumented INNEX FIXUC ultra-congruent implant design. Besides the load data of the TKR, *Stan* provides joint kinematics and pose of the corresponding femoral and tibial coordinate systems.

In the following, the steps required to transfer the standardised load cases to different TKR designs using the VIVO™ joint simulator are described. First, the scaling of the load case had to be defined. Bergmann et al.^44^ proposed three different load scales: AVER75, HIGH100 and PEAK100 when transmitting telemetric measured data. AVER75, which imitates a body weight of 75 kg, was chosen for this study as a compromise between physiological loads and to avoid the physical limits of the joint simulator being exceeded^45^.

**Fig. 4:**
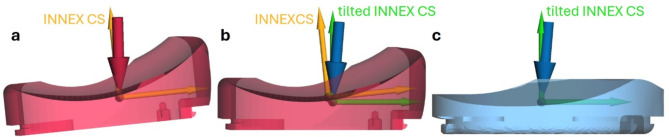
Adjusting the coordinate system. (**a**) Tibial insert of the INNEX TKR (light red) with INNEX coordinate system (CS) (orange) and exemplarily visualised resultant force (dark red) as supplied by the CAMS dataset; (**b**) Tibial insert of the INNEX knee tilted by 6.5° tibial slope with respect to the INNEX CS (orange), tilted INNEX CS (green) and tilted resultant force (dark blue); (**c**) Tibial insert of the P.F.C. Sigma (light blue) with tilted INNEX CS (green) and tilted resultant force (dark blue). The images were generated with the multibody software Simpack (V 2022x, Dassault Systèmes, Vélizy-Villacoublay, France, available from: https://www.3ds.com/) and edited with Paint.NET (V5.0.1, Washington State University, Pullman, WA, USA and Microsoft Corporation, Redmond, WA, USA, https://www.getpaint.net/).

One fundamental question of this study was the plausibility of a transfer of dynamics to another TKR design. Further, a right cruciate-retaining design (P.F.C.^®^ Sigma, DePuy Synthes, Raynham, MA, USA) was used for testing. Furthermore, an individual ligament apparatus was used for the standardised load case, which needed a precise definition of the implant pose relative to the surrounding soft tissue structures. According to Dreyer et al.^20^ the standardised loads and kinematics referred to an implant coordinate system (in the following referred to as INNEX coordinate system) need to be adjusted when testing other TKR designs. To achieve a precise definition of the coordinate systems, both TKR, namely the INNEX, as well as the P.F.C. Sigma, were virtually implanted using GEOMAGIC Studio v13 (3D Systems, Rock Hill, SC, USA) with their respective implant coordinate systems and a multibody simulation framework (SIMPACK v2022, Dassault Systèmes, Vélizy-Villacoublay, France) of a tibiofemoral joint with a defined ligament apparatus. This was possible as the CAMS dataset provided the implant CAD data, including the INNEX coordinate system. The patients equipped with the INNEX TKR within the CAMS study were implanted with an average posterior tibial slope of 8.5°^20^, which is higher compared to other designs such as the P.F.C. Sigma (slope up to 5° is generally appropriate according to the surgical technique^46^). The loads, particularly the anterior-posterior forces, can lead to unphysiological loads for unconstrained implants. Therefore, it was assumed that the implants (INNEX and P.F.C. Sigma) were implanted with the same slope, and the functional surfaces were matched, resulting in a slope correction of 6.5° and shifts of the coordinate system of up to 1 mm for the P.F.C. Sigma. Figure 4 shows the tibial inserts of both implants with their corresponding coordinate systems as they were virtually implanted in the tibial bone and an exemplarily chosen resultant force of the load case *downhill walking* and both the INNEX coordinate system (A) and the tilted INNEX coordinate system (C) with the corresponding load.

As depicted in Fig. 4, the kinematics and kinetics of the CAMS dataset were transformed from the INNEX coordinate system (orange) to the P.F.C. Sigma coordinate system (light blue). This led to a change in the forces and particularly the moments due to the shift of both coordinate system origins.

As the VIVO™ joint simulator is based on the Grood and Suntay convention, the newly obtained kinematics and kinetics for the P.F.C. Sigma in Cartesian coordinates had to be converted^26^. For the experimental tests in this study, the flexion-extension and external-internal rotation were position-controlled. In contrast, the anterior-posterior, medial-lateral and inferior-superior directions and the abduction-adduction rotation were force- and moment-controlled.

Concerning the control strategy, load cases on the VIVO™ joint simulator can be controlled using proportional–integral–derivative (PID) controller or iterative learning control (ILC)^26^. The ILC was chosen for this study as it minimises the control error with increasing cycles. For this purpose, the five load cases had to be periodised, i.e. the start and end values had to be harmonised. This was achieved by extending the respective waveforms by 15% through extrapolation.

**Fig. 5:**
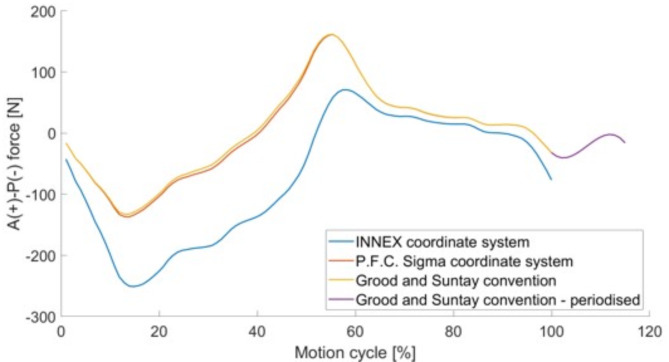
Preparation of force data for integration into the joint simulator. Anterior (A)-posterior (P) force shown for the INNEX coordinate system, the P.F.C. Sigma coordinate system and the Grood and Suntay convention. The periodised data in the Grood and Suntay convention were transferred to the VIVO™ joint simulator.

Exemplarily, the anterior-posterior force is shown for the INNEX coordinate system, for the P.F.C. Sigma coordinate system, for the P.F.C. Sigma coordinate system in the Grood and Suntay convention and the periodised manner in Fig. 5.

### Implementation of the virtual ligament apparatus

**Fig. 6:**
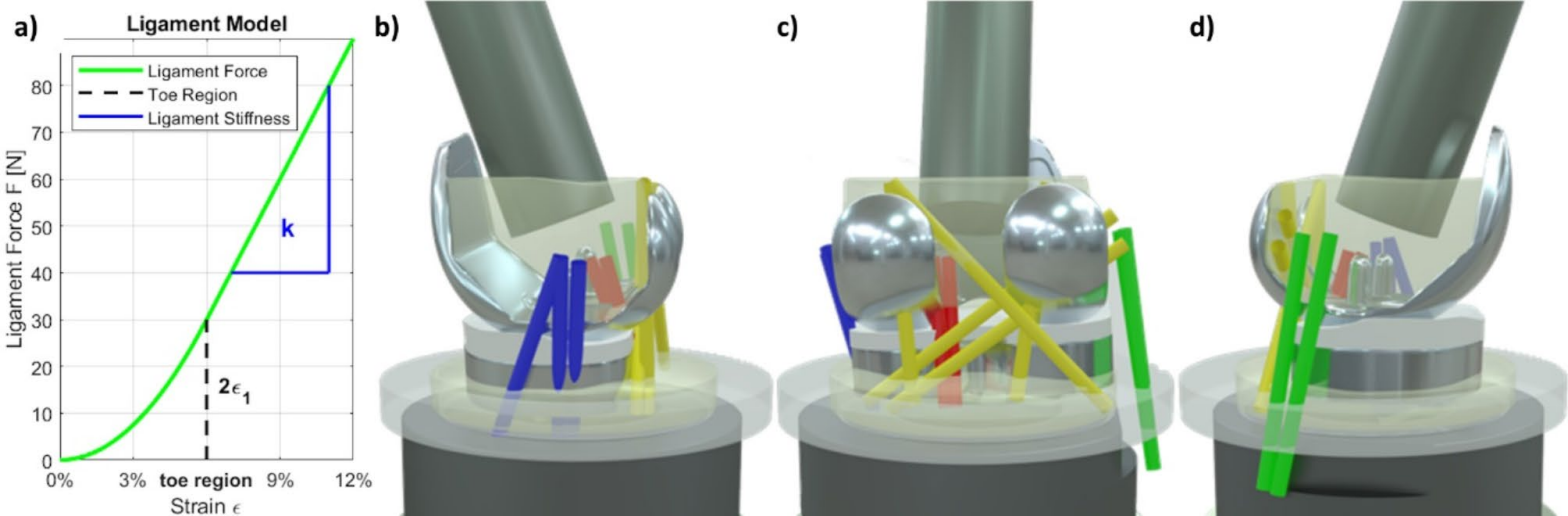
Illustration of the virtual ligament apparatus used in the experimental test setup with an exemplary force-strain curve of the ligament model (**a**). Virtual ligaments considered during experimental tests for the total knee replacement viewed from medial (**b**), posterior (**c**) and lateral (**d**) can be grouped in capsular structures (yellow), medial structures (blue), lateral structures (green) and the posterior cruciate ligament (red). Note that the bones are not depicted. The diagram was generated using MATLAB software (V R2022b, MathWorks Inc., MA, USA, https://de.mathworks.com/products/new_products/release2022b.html), and the images (b-d) of the test setup were rendered using the open-source software Blender (V 4.0, Blender Foundation, Amsterdam, Netherlands, https://download.blender.org/release/).

As described, the relative position and orientation of the implants to each other using the VIVO™ joint simulator is described once by defining a “reference pose”^26^. To ensure that the implants are correctly aligned when setting the reference pose, both the femoral component and the tibial component were previously embedded in self-curing polymer (Rencast^®^ FC 52/53 Isocyanat and FC 53 Polyol, Huntsman Advanced Materials GmbH, Bergkamen, Germany) in a definite position and orientation using 3D-printed templates^19^. The experimental test setup is visualised in Fig. 6. According to Kleist et al.^19^, the VIVO™ joint simulator has a compliance that leads to an unwanted misinterpretation of the ligament forces. To counteract this, it was proposed that the implant should be loaded with the mean compression force of the respective load case when setting the reference position. This led to initial compression forces ranging from − 842 N for the *squat* motion to − 1553 N for *stairs down*. The previously prepared standardised load cases were transferred to the VIVO™ joint simulator as joint reaction forces. These forces summated the contact forces/moments measured in the force transducer and the virtual ligament forces/moments. As ligament forces (tension) were directed in the opposite direction to the contact forces, the applied load on the implant increased with higher ligament forces^26^.

The VIVO™ joint simulator is able to virtually consider ligament-induced forces and moments during load cases depending on joint pose according to Blankevoort et al.^47^ and Wismans et al.^48^. For our present study, 12 ligament strands were considered, grouped into capsular structures, medial ligament structures, lateral structures and the posterior cruciate ligament (PCL). Input parameters for each strand were origin and insertion points, ligament stiffness and reference strain. The origin point referred to the femoral attachment point, the insertion point was the attachment point on the tibia and the reference strain was the strain to a known joint pose (usually full extension for the tibiofemoral joint). According to Wismans et al.^48^ and Blankevoort et al.^47^, each ligament strand is described by a non-linear force-strain behaviour. This force is acting between origin and insertion point and depends on the current ligament strain $\:\epsilon\:$ which can be determined using the reference length $\:{l}_{r}$ (length of the ligament in the reference pose), the reference strain $\:{\epsilon\:}_{r}$ (pre-tension of the ligament in the reference pose) and the current ligament length $\:l\:$ (i.e. current distance between insertion and origin point):

1 $$\:\epsilon\:=\:\frac{{\epsilon\:}_{r}*l}{{l}_{r}}*100\%$$

The reference pose describes an arbitrary posture for which the actual strain is known. In case of the knee joint, this is usually the fully extended position. No force is applied for strains below 0%. Within the initial elongation phase, referred to as the toe region $\:\epsilon\:=2{\epsilon\:}_{1}\:$(with $\:{\epsilon\:}_{1}$ set at 3% in the VIVO™ joint simulator), the ligament force increases quadratically with strain. Beyond this threshold, the force grows linearly with further strain. The ligament force depends on the ligament stiffness k in both the linear and quadratic regions.

2 $$f = \left\{ {\begin{array}{*{20}l} 0 \hfill & {\varepsilon < 0} \hfill \\ {\frac{1}{4}\frac{{k\varepsilon ^{2} }}{{\varepsilon _{l} }}} \hfill & {0 \le \varepsilon \le 2\varepsilon _{l} ,} \hfill \\ {k(\varepsilon - \varepsilon l)} \hfill & {\varepsilon > 2\varepsilon _{l} } \hfill \\ \end{array} } \right.$$

The ligament parameters were obtained from the literature^1,26^ and are included in the supplementary information. The locations of the ligaments are visualised in Fig. 6.

### Experimental testing

#### Transfer of standardised load cases

After mounting the implants and setting the reference position, 1 ml of silicone oil (Silikonöl Typ 350, Caesar & Loretz GmbH, Hilden, Germany) was evenly distributed over the tibial condyles as a lubricant. The respective load case and the ligament apparatus to be tested were then loaded, and the test frequency corresponding to the load case was set. All load cases were performed in real-time. *Level walking* was the fastest load case with a test frequency of 0.625 Hz, while *squat* was the slowest with a test frequency of 0.2 Hz. A total of 300 cycles were tested for each trial to ensure the convergence of the iterative control algorithm^26^, with the last five cycles being averaged for the subsequent evaluation. The following values were extracted with the reference ligament apparatus, i.e. the inferior-superior force (axial load), the anterior-posterior force, the anterior-posterior translation, the flexion-extension, and the abduction-adduction angle were analysed over the different movement cycles.

#### Tibiofemoral contact areas

Moreover, the contact surfaces on the tibial insert in different load cases were compared. For this purpose, the tibial surface was sprayed with sublimating scanning spray (Reflecon Tarnish 11, MR Chemie GmbH, Unna, Germany). The five load cases were then run three times in a row purely kinematically (0.05 Hz) to determine the contact areas afterwards utilizing a digital microscope (VHX-6000, Keyence Deutschland GmbH, Neu-Isenburg, Germany).

#### Variation of the ligament apparatus

Additionally, an analysis regarding the influence of the soft tissue situation on the resulting joint kinematics and kinetics using standardised load cases was performed. Therefore, the experiments were repeated under three additional soft tissue conditions: doubling the lateral structures’ stiffnesses, doubling the medial structures’ stiffnesses and resecting the posterior cruciate ligament, demonstrating the sensitivity of our approach as it was done in the literature^18,19^. During this analysis, the effects on the anterior-posterior translation and forces, as well as the abduction-adduction angles, were of particular interest. In contrast to the other investigations, the results were analysed with respect to the flexion angle.

Data from both experimental and numerical studies on the load cases considered in present work were published^34,40,49–53^. However, due to the lack of information regarding the exact position of the defined coordinate systems and different load profiles compared to the *Stan* dataset, a comparison with the data of these studies is not practical. Instead, the *Stan* dataset was processed kinematically and dynamically in the same coordinate system as well as according to the Grood and Suntay convention (as defined for the VIVO™ joint simulator). The datasets are shown in the supplementary results. This approach enabled a subsequent comparison between in vivo data derived with the INNEX total knee system^20^ and in vitro data from the P.F.C. Sigma.

## Electronic supplementary material

Below is the link to the electronic supplementary material.

Supplementary Material 1

## Data Availability

The authors confirm that the data supporting the findings of this study are available within the article.

## References

[1] Kebbach, M. et al. Musculoskeletal multibody simulation analysis on the impact of patellar component design and positioning on joint dynamics after unconstrained total knee arthroplasty. *Mater. (Basel Switz.)* **13** (2020). 10.3390/ma13102365 PMC7287668 32455672

[2] Williams, J. L. & Gomaa, S. T. in *Computational Biomechanics for Medicine*, edited by A. Wittek, K. Miller & P. M. Nielsen 157–168 (Springer, 2013).

[3] Asseln, M., Grothues, S. A. G. A. & Radermacher, K. Relationship between the form and function of implant design in total knee replacement. *J. Biomech.* **119**, 110296 (2021). 33676270 10.1016/j.jbiomech.2021.110296

[4] Hettich, G., Weiß, J. B., Wünsch, B. & Grupp, T. M. Finite element analysis for pre-clinical testing of custom-made knee implants for complex reconstruction surgery. *Appl. Sci.* **12**, 4787 (2022).

[5] Kwon, O. R. et al. Biomechanical comparison of fixed- and mobile-bearing for unicomparmental knee arthroplasty using finite element analysis. *J. Orthop. Res. Off. Publ. Orthop. Res. Soc.* **32**, 338–345 (2014). 10.1002/jor.22499 24122942

[6] ISO International Organization for Standardization. *Implants for surgery - Wear of Total knee-joint prostheses - Part 1: Loading and Displacement Parameters for wear-testing Machines with Load Control and Corresponding Environmental Conditions for Test* (Beuth Verlag GmbH, 2009).

[7] Schütz, P. et al. Knee implant kinematics are task-dependent. *J. R. Soc. Interface*. **16**, 20180678 (2019). 30958178 10.1098/rsif.2018.0678 PMC6408358

[8] Taylor, W. R. et al. A comprehensive assessment of the musculoskeletal system: The CAMS-Knee data set. *J. Biomech.* **65**, 32–39 (2017). 29037443 10.1016/j.jbiomech.2017.09.022

[9] Reinders, J. et al. Wear testing of moderate activities of daily living using in vivo measured knee joint loading. *PLoS ONE*. **10**, e0123155 (2015). 25811996 10.1371/journal.pone.0123155 PMC4374780

[10] Schwiesau, J. et al. Knee wear simulation under conditions of highly demanding daily activities–influence on an unicompartmental fixed bearing knee design. *Med. Eng. Phys.* **35**, 1204–1211 (2013). 23380535 10.1016/j.medengphy.2012.12.015

[11] Fitzpatrick, C. K. et al. Validation of a new computational 6-DOF knee simulator during dynamic activities. *J. Biomech.* **49**, 3177–3184 (2016). 27545078 10.1016/j.jbiomech.2016.07.040

[12] Thomeer, L. et al. Six-Degree-of-Freedom tibiofemoral and patellofemoral joint motion during activities of daily living. *Ann. Biomed. Eng.* **49**, 1183–1198 (2021). 33094419 10.1007/s10439-020-02646-2

[13] Kozanek, M. et al. Tibiofemoral kinematics and condylar motion during the stance phase of gait. *J. Biomech.* **42**, 1877–1884 (2009). 19497573 10.1016/j.jbiomech.2009.05.003 PMC2725209

[14] Maag, C., Cracaoanu, I., Langhorn, J. & Heldreth, M. Total knee replacement wear during simulated gait with mechanical and anatomic alignments. *Proc. Inst. Mech. Eng. H*. **235**, 515–522 (2021). 33522419 10.1177/0954411921991269

[15] Dreyer, M. J. et al. Experimental and computational evaluation of knee implant wear and creep under in vivo and ISO boundary conditions. *Biomed. Eng. Online*. **23**, 130 (2024). 39716270 10.1186/s12938-024-01321-0 PMC11664841

[16] Vakili, S., Lanting, B., Getgood, A. & Willing, R. *Development of Multi-Bundle Virtual Ligaments to Simulate Knee Mechanics after Total Knee Arthroplasty* (2022). 10.1115/1.4062421 37216311

[17] Vakili, S., Lanting, B., Getgood, A. & Willing, R. Comparison of the kinematics and laxity of total knee arthroplasty bearing designs stabilized with Specimen-Specific virtual ligaments on a joint motion simulator. *J. Biomech. Eng.* **146** (2024). 10.1115/1.4064621 38529555

[18] Kebbach, M. et al. Effect of surgical parameters on the Biomechanical behaviour of bicondylar total knee endoprostheses—A robot-assisted test method based on a musculoskeletal model. *Sci. Rep.* **9**, 14504 (2019). 31601894 10.1038/s41598-019-50399-3 PMC6787084

[19] Kleist, E. et al. Impact of structural compliance of a six degree of freedom joint simulator on virtual ligament force calculation in total knee endoprosthesis testing. *Life* **14**, 531 (2024). 38672801 10.3390/life14040531 PMC11050787

[20] Dreyer, M. J. et al. European society of biomechanics S.M. Perren award 2022: Standardized tibio-femoral implant loads and kinematics. *J. Biomech.* **141**, 111171 (2022). 35803037 10.1016/j.jbiomech.2022.111171

[21] Willing, R. & Walker, P. S. Measuring the sensitivity of total knee replacement kinematics and laxity to soft tissue imbalances. *J. Biomech.* **77**, 62–68 (2018). 30078414 10.1016/j.jbiomech.2018.06.019

[22] Saeidi, M. et al. Preliminary Biomechanical cadaver study investigating a new load-sharing knee implant. *J. Exp. Orthop.* **8**, 61 (2021). 10.1186/s40634-021-00379-2 PMC8364593 34392435

[23] Clary, C. & Maletsky, L. In *Mechanical Testing of Orthopaedic Implants* 207–229 (Elsevier, 2017).

[24] Bauer, L. et al. Does posterior tibial slope influence knee kinematics in medial stabilized TKA? *J. Clin. Med.* **11** (2022). 10.3390/jcm11226875 PMC9698522 36431352

[25] Grood, E. S. & Suntay, W. J. A joint coordinate system for the clinical description of three-dimensional motions: Application to the knee. *J. Biomech. Eng.* **105**, 136–144 (1983). 6865355 10.1115/1.3138397

[26] Henke, P., Ruehrmund, L., Bader, R. & Kebbach, M. Exploration of the advanced VIVOTM joint simulator: An In-Depth analysis of opportunities and limitations demonstrated by the artificial knee joint. *Bioeng. (Basel Switz.)* **11** (2024). 10.3390/bioengineering11020178 PMC10886281 38391664

[27] Chaiyakit, P. & Dokkhum, P. Posterior cruciate ligament resection and varus correction in total knee arthroplasty: A study using Computer-Assisted surgery. *Arthroplast. Today*. **13**, 176–180 (2022). 35097174 10.1016/j.artd.2021.11.007 PMC8783108

[28] Demura, T., Demura, S. & Shin, S. Comparison of gait properties during level walking and stair ascent and descent with varying loads. *Health* **02**, 1372–1376 (2010).

[29] Gascoyne, T. et al. In vivo wear measurement in a modern total knee arthroplasty with model-based radiostereometric analysis. *Bone Joint J.* **101-B**, 1348–1355 (2019). 31674253 10.1302/0301-620X.101B11.BJJ-2018-1447.R2

[30] Heller, M. O., Taylor, W. R., Perka, C. & Duda, G. N. The influence of alignment on the musculo-skeletal loading conditions at the knee. *Langenbeck’s Arch. Surg.* **388**, 291–297 (2003). 10.1007/s00423-003-0406-2 13680238

[31] Schwarzkopf, R., Scott, R. D., Carlson, E. M. & Currier, J. H. Does increased topside conformity in modular total knee arthroplasty lead to increased backside wear? *Clin. Orthop. Relat. Res.* **473**, 220–225 (2015). 24777725 10.1007/s11999-014-3648-0 PMC4390912

[32] Shelburne, K. B. & Pandy, M. G. A dynamic model of the knee and lower limb for simulating rising movements. *Comput. Methods Biomech. BioMed. Eng.* **5**, 149–159 (2002). 10.1080/10255840290010265 12186724

[33] Markolf, K. L., Mensch, J. S. & Amstutz, H. C. Stiffness and laxity of the knee–the contributions of the supporting structures. A quantitative in vitro study. *J. Bone Joint Surg. Am.* **58**, 583–594 (1976). 946969

[34] Fitzpatrick, C. K., Baldwin, M. A., Clary, C. W., Maletsky, L. P. & Rullkoetter, P. J. Evaluating knee replacement mechanics during ADL with PID-controlled dynamic finite element analysis. *Comput. Methods Biomech. BioMed. Eng.* **17**, 360–369 (2014). 10.1080/10255842.2012.684242 22687046

[35] Montgomery, L., McGale, J., Lanting, B. & Willing, R. Biomechanical analysis of ligament modelling techniques in TKA knees during laxity tests using a virtual joint motion simulator. *Comput. Methods Biomech. BioMed. Eng.*, 1–13 (2023). 10.1080/10255842.2023.2256925 37703067

[36] Hull, M. L. Coordinate system requirements to determine motions of the tibiofemoral joint free from kinematic crosstalk errors. *J. Biomech.* **109**, 109928 (2020). 32807309 10.1016/j.jbiomech.2020.109928

[37] Ortigas Vásquez, A. et al. A frame orientation optimisation method for consistent interpretation of kinematic signals. *Sci. Rep.* **13**, 9632 (2023). 37316703 10.1038/s41598-023-36625-z PMC10267167

[38] Tischer, T. et al. Patella height influences patellofemoral contact and kinematics following cruciate-retaining total knee replacement. *J. Orthop. Res. Off. Publ. Orthop. Res. Soc.* **41**, 793–802 (2023). 10.1002/jor.25425 35949157

[39] Kebbach, M. et al. Biomechanical assessment of Mobile-Bearing total knee endoprostheses using musculoskeletal simulation. *Appl. Sci.* **12**, 182 (2022).

[40] Kebbach, M. et al. Computer-based analysis of different component positions and insert thicknesses on tibio-femoral and patello-femoral joint dynamics after cruciate-retaining total knee replacement. *Knee* **40**, 152–165 (2023). 36436384 10.1016/j.knee.2022.11.010

[41] Wu, G. et al. ISB recommendation on definitions of joint coordinate system of various joints for the reporting of human joint motion–part I: Ankle, hip, and spine. International society of biomechanics. *J. Biomech.* **35**, 543–548 (2002). 11934426 10.1016/s0021-9290(01)00222-6

[42] Yang, T. et al. 6DOF knee kinematic alterations due to increased load levels. *Front. Bioeng. Biotechnol.* **10**, 927459 (2022). 36213071 10.3389/fbioe.2022.927459 PMC9533867

[43] Andreassen, T. E. et al. Apparatus for in vivo knee laxity assessment using High-Speed stereo radiography. *J. Med. Devices*. **15**, 41004 (2021). 10.1115/1.4051834 PMC8546959 34721751

[44] Bergmann, G. et al. Standardized loads acting in knee implants. *PLoS ONE*. **9**, e86035 (2014). 24465856 10.1371/journal.pone.0086035 PMC3900456

[45] AMTI Force and Motion. VivoControl Software Technical Reference Manual. Version 1.0.0. (2015).

[46] DePuy & Synthes *P.F.C. SIGMA KNEE SYSTEMS. Surgical Technique*.

[47] Blankevoort, L., Kuiper, J. H., Huiskes, R. & Grootenboer, H. J. Articular contact in a three-dimensional model of the knee. *J. Biomech.* **24**, 1019–1031 (1991). 1761580 10.1016/0021-9290(91)90019-j

[48] Wismans, J., Veldpaus, F., Janssen, J., Huson, A. & Struben, P. A three-dimensional mathematical model of the knee-joint. *J. Biomech.* **13**, 677–685 (1980). 7419534 10.1016/0021-9290(80)90354-1

[49] Schellenberg, F. et al. Evaluation of the accuracy of musculoskeletal simulation during squats by means of instrumented knee prostheses. *Med. Eng. Phys.* **61**, 95–99 (2018). 30282587 10.1016/j.medengphy.2018.09.004

[50] Freed, R. D. et al. Are instrumented knee forces representative of a larger population of Cruciate-Retaining total knee arthroplasties? *J. Arthroplast.* **32**, 2268–2273 (2017). 10.1016/j.arth.2017.01.054 PMC5469705 28262455

[51] Lu, Y. et al. A comparative study on loadings of the lower extremity during deep squat in Asian and Caucasian individuals via opensim musculoskeletal modelling. *Biomed. Res. Int.* **2020**, 1–10 (2020).

[52] Pelegrinelli, A. R. M., Catelli, D. S., Kowalski, E., Lamontagne, M. & Moura, F. A. Comparing three generic musculoskeletal models to estimate the tibiofemoral reaction forces during gait and sit-to-stand tasks. *Med. Eng. Phys.* **122**, 104074 (2023). 38092489 10.1016/j.medengphy.2023.104074

[53] Trepczynski, A. et al. Tibio-Femoral contact force distribution is not the only factor governing Pivot location after total knee arthroplasty. *Sci. Rep.* **9**, 182 (2019). 30655583 10.1038/s41598-018-37189-z PMC6336768

