# Supplementary material for: Transfer of patients’ tibiofemoral kinematics and loads to a six-degree-of-freedom (6-DOF) joint simulator under consideration of virtual ligaments
Source: Sci Rep. 2025 Mar 27;15:10512. doi: 10.1038/s41598-025-95400-4 (PMC11947412; doi:10.1038/s41598-025-95400-4)
Supplement: Supplementary file 1 — Supplementary Material 1 [file 41598_2025_95400_MOESM1_ESM.docx]

**Supplementary Information**


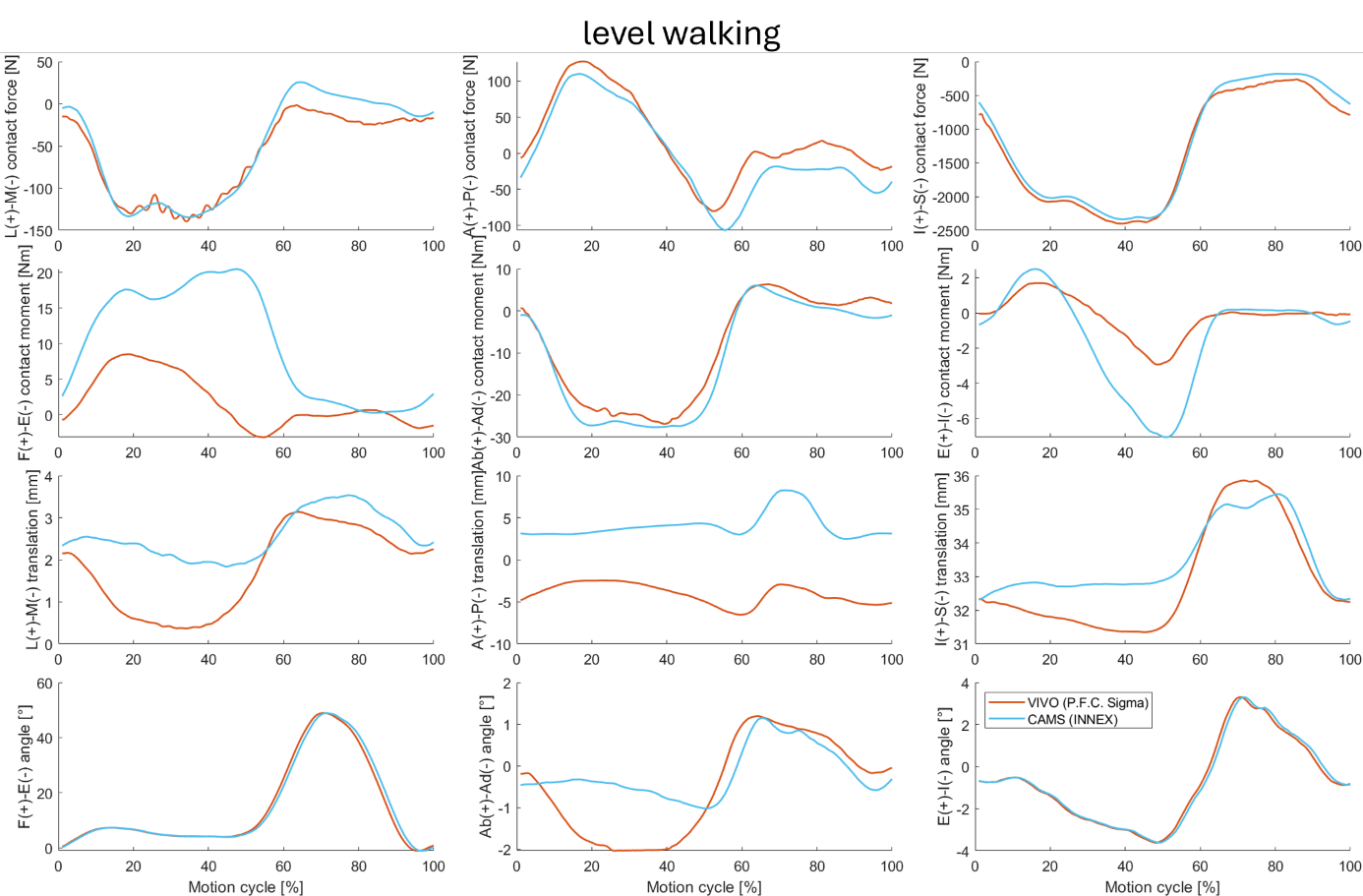


Supplementary Figure 1: Kinematics and kinetics for the VIVO^TM^ joint simulator tests (P.F.C. Sigma) and the CAMS knee dataset (INNEX) of the load case *level walking*.


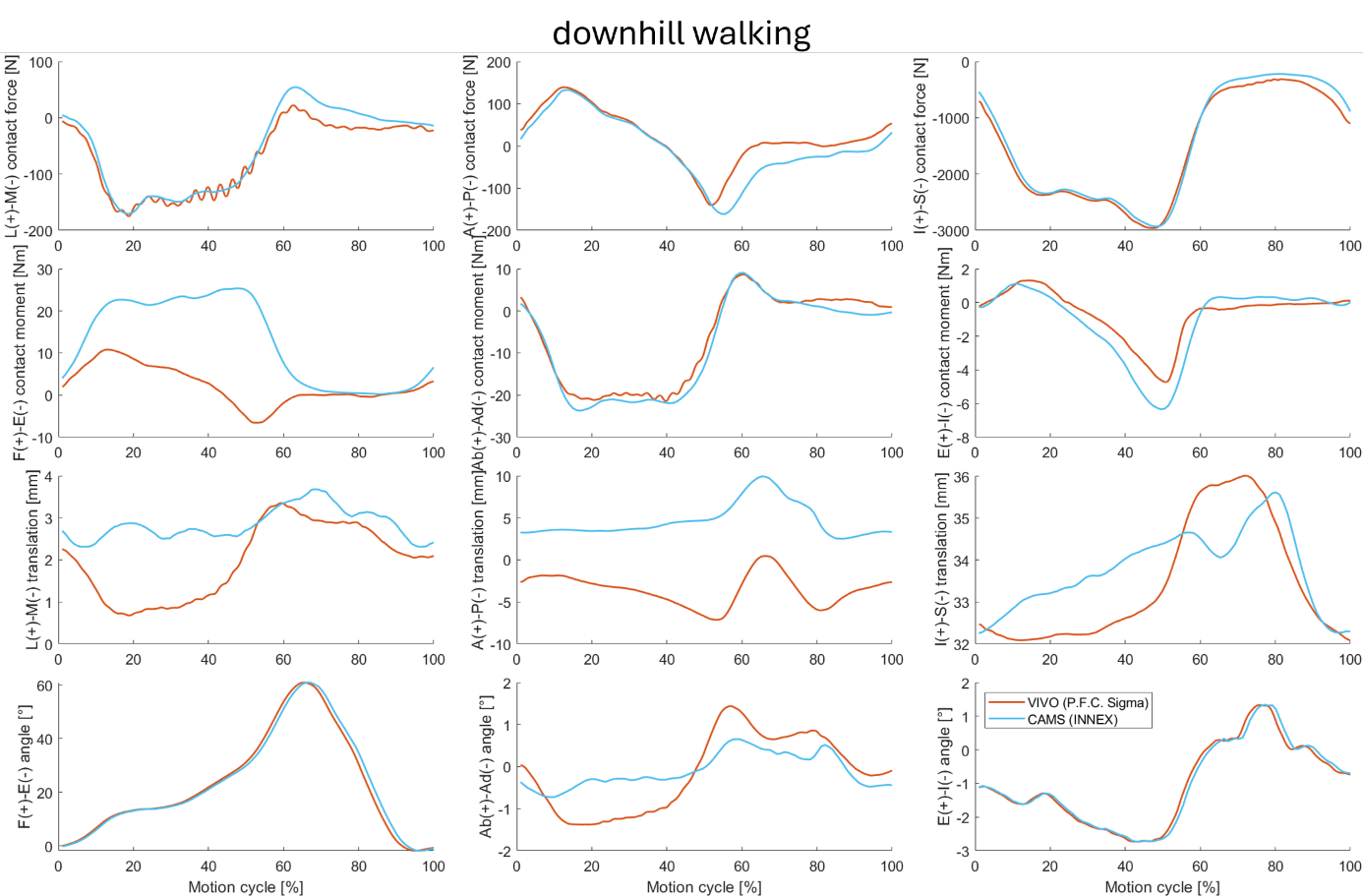


Supplementary Figure 2: Kinematics and kinetics for the VIVO^TM^ joint simulator tests (P.F.C. Sigma) and the CAMS knee dataset (INNEX) of the load case *downhill walking*.


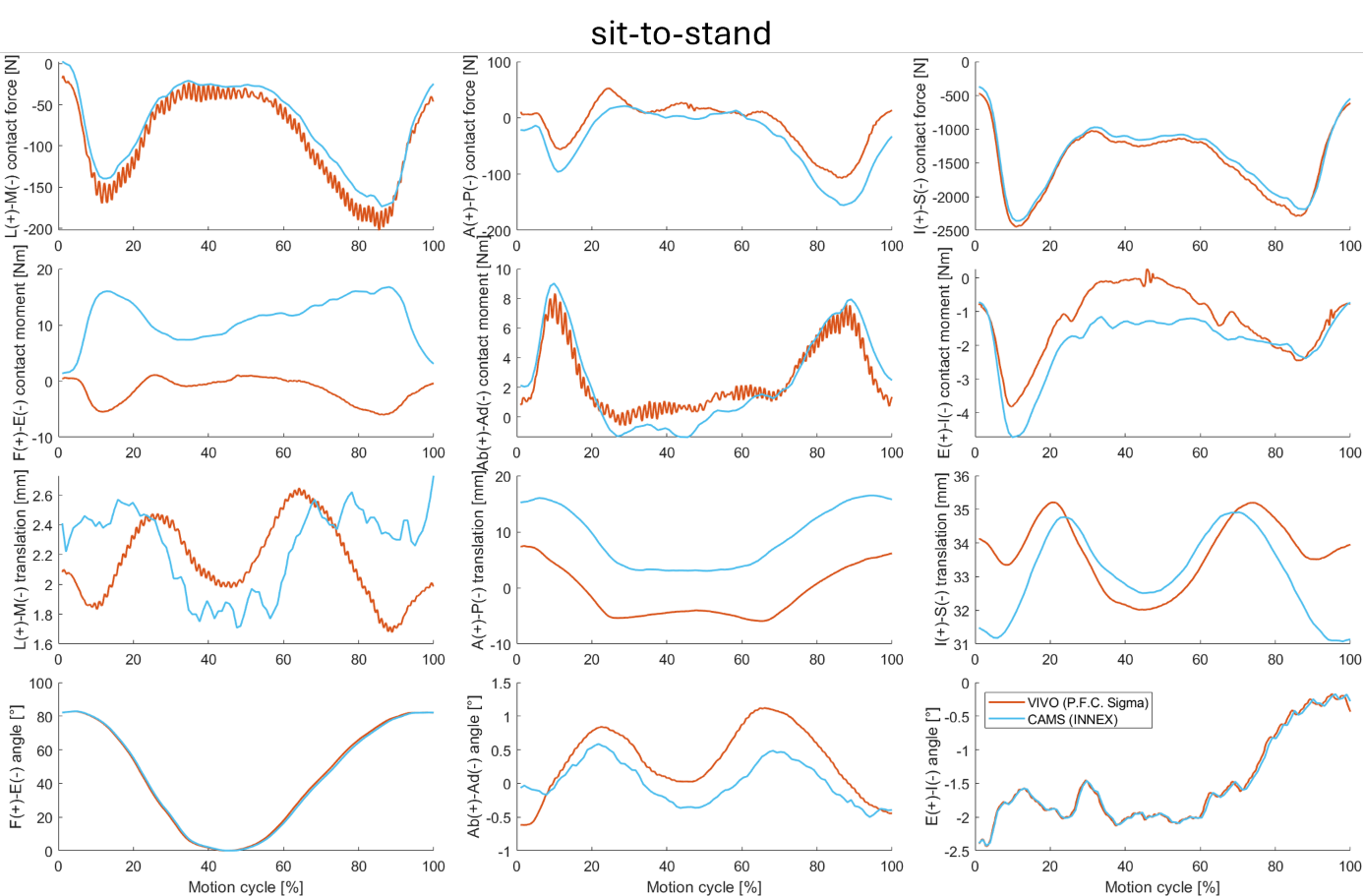


Supplementary Figure 3: Kinematics and kinetics for the VIVO^TM^ joint simulator tests (P.F.C. Sigma) and the CAMS knee dataset (INNEX) of the load case *sit-to-stand*.


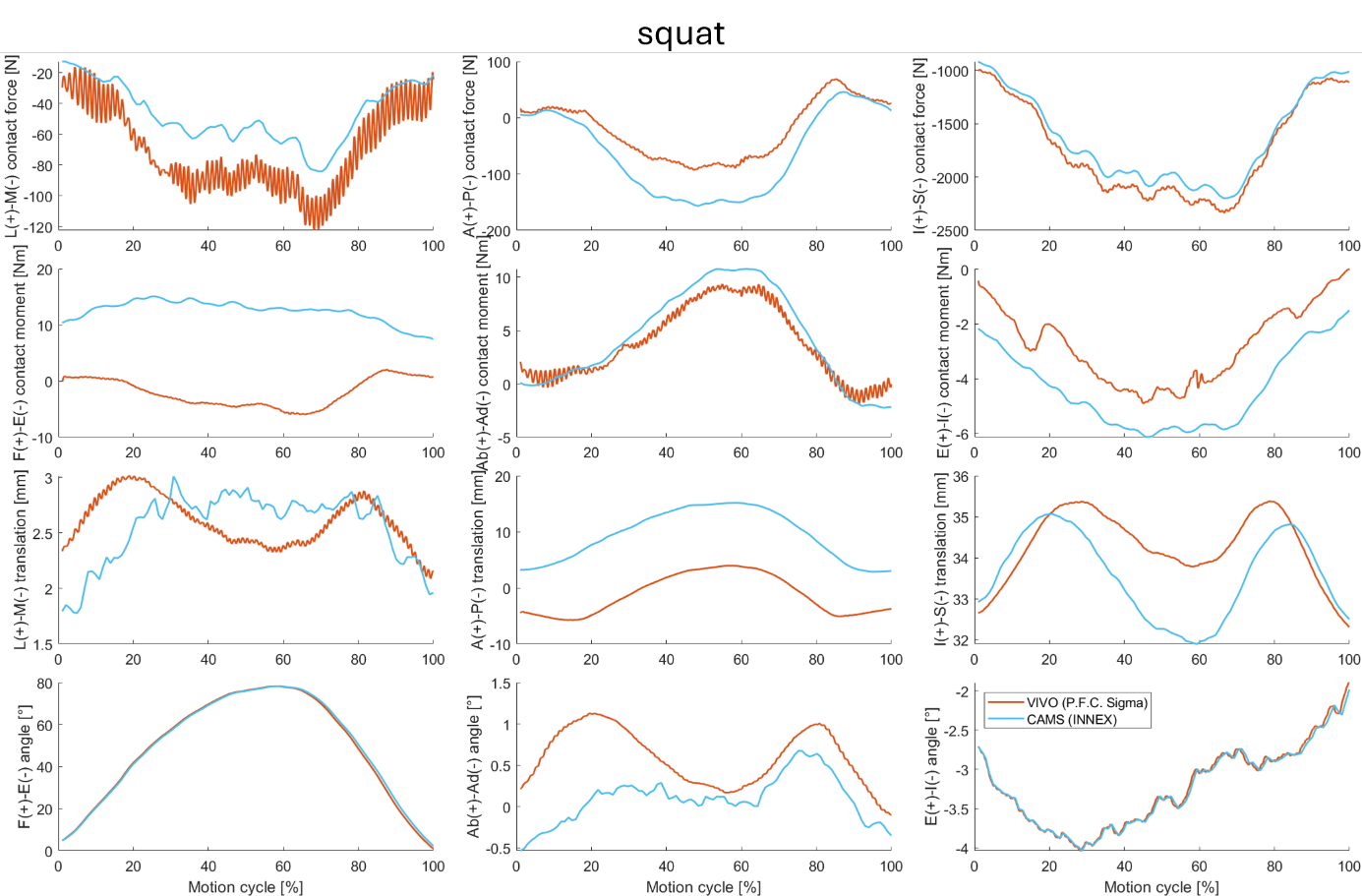


Supplementary Figure 4: Kinematics and kinetics for the VIVO^TM^ joint simulator tests (P.F.C. Sigma) and the CAMS knee dataset (INNEX) of the load case *squat*.


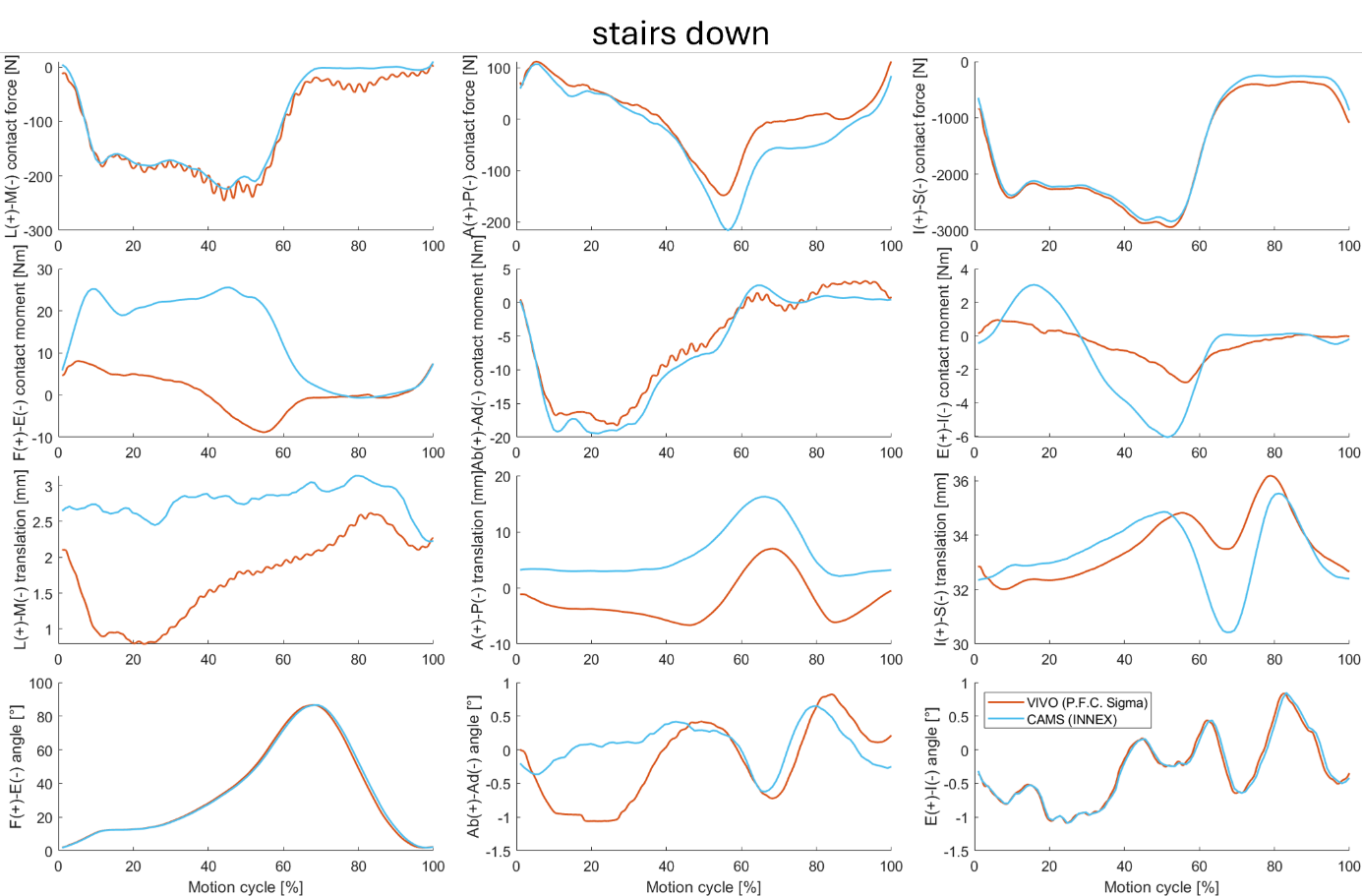


Supplementary Figure 5: Kinematics and kinetics for the VIVO^TM^ joint simulator tests (P.F.C. Sigma) and the CAMS knee dataset (INNEX) of the load case *stairs down*.
